# Prediction of Human Induced Pluripotent Stem Cell Cardiac Differentiation Outcome by Multifactorial Process Modeling

**DOI:** 10.3389/fbioe.2020.00851

**Published:** 2020-07-23

**Authors:** Bianca Williams, Wiebke Löbel, Ferdous Finklea, Caroline Halloin, Katharina Ritzenhoff, Felix Manstein, Samira Mohammadi, Mohammadjafar Hashemi, Robert Zweigerdt, Elizabeth Lipke, Selen Cremaschi

**Affiliations:** ^1^Department of Chemical Engineering, Auburn University, Auburn, AL, United States; ^2^Leibniz Research Laboratories for Biotechnology and Artificial Organs (LEBAO), Department of Cardiothoracic, Transplantation and Vascular Surgery, Hannover Medical School, Hanover, Germany

**Keywords:** machine learning, classification, feature selection, human induced pluripotent stem cells, cardiomyocytes, directed differentiation, bioreactor, cell production

## Abstract

Human cardiomyocytes (CMs) have potential for use in therapeutic cell therapy and high-throughput drug screening. Because of the inability to expand adult CMs, their large-scale production from human pluripotent stem cells (hPSC) has been suggested. Significant improvements have been made in understanding directed differentiation processes of CMs from hPSCs and their suspension culture-based production at chemically defined conditions. However, optimization experiments are costly, time-consuming, and highly variable, leading to challenges in developing reliable and consistent protocols for the generation of large CM numbers at high purity. This study examined the ability of data-driven modeling with machine learning for identifying key experimental conditions and predicting final CM content using data collected during hPSC-cardiac differentiation in advanced stirred tank bioreactors (STBRs). Through feature selection, we identified process conditions, features, and patterns that are the most influential on and predictive of the CM content at the process endpoint, on differentiation day 10 (dd10). Process-related features were extracted from experimental data collected from 58 differentiation experiments by feature engineering. These features included data continuously collected online by the bioreactor system, such as dissolved oxygen concentration and pH patterns, as well as offline determined data, including the cell density, cell aggregate size, and nutrient concentrations. The selected features were used as inputs to construct models to classify the resulting CM content as being “*sufficient*” or “*insufficient*” regarding pre-defined thresholds. The models built using random forests and Gaussian process modeling predicted *insufficient* CM content for a differentiation process with 90% accuracy and precision on dd7 of the protocol and with 85% accuracy and 82% precision at a substantially earlier stage: dd5. These models provide insight into potential key factors affecting hPSC cardiac differentiation to aid in selecting future experimental conditions and can predict the final CM content at earlier process timepoints, providing cost and time savings. This study suggests that data-driven models and machine learning techniques can be employed using existing data for understanding and improving production of a specific cell type, which is potentially applicable to other lineages and critical for realization of their therapeutic applications.

## Introduction

The heart is one of the least regenerative organs in the body; therefore, when disease or damage occurs to the myocardium, native cardiac muscle cells, cardiomyocytes (CMs), are replaced with fibrotic scar tissue. Recent work has shown that CMs can be derived from human pluripotent stem cells (hPSCs; including embryonic and induced pluripotent stem cells hESC and hiPSC, respectively) at more chemically defined conditions (Lian et al., 2012; Burridge et al., 2014) and that these cells have immense therapeutic potential (Chong et al., 2014). However, due to the large number of patients that suffer from cardiovascular disease along with the vast number of cells presumably needed for a therapeutic effect, scalable production of CMs in a consistent and reproducible manner is critical for the clinical translation and success of these treatments. Proof-of-concept for the production and directed cardiac differentiation of hPSCs in industry-compatible stirred tank bioreactors (STBRs) has been demonstrated (Kempf et al., 2014, 2015; Kropp et al., 2016; Halloin et al., 2019). However, the experimental development, optimization and upscaling of this complex, multifactorial process is time consuming, costly, and despite the recent success, still highly variable. The multifaceted interplay of numerous cellular, physiological, and mechanical parameters including hPSC expansion at the pluripotent state, impacts their directed differentiation, leading to challenges in establishing robust protocols for their efficient lineage-specific, i.e., cardiac, differentiation in bioreactors. The resulting variability in endpoint cell purity, or CM content, together with time constraints, CMs’ phenotype and maturity impede commercial production and progress to clinical translation. This also precludes the use of hPSC-CMs for other mass applications, including high-throughput screenings for drug development and safety pharmacology (Fonoudi et al., 2015; Sun and Nunes, 2017; Machiraju and Greenway, 2019) and faster progress in cardiac tissue engineering (Kensah et al., 2013).

The potential of hPSCs for unlimited proliferation *in vitro* and their ability to differentiate into derivatives of the three germ layers (endo-, ecto-, and mesoderm) paved the way toward clinically relevant mass production of specific progenies required for disease-specific therapies, including CMs (Hazeltine et al., 2013). Cardiomyocyte differentiation is inherently complex; cardiac differentiation from hPSCs occurs through specific stages, including early primitive-streak-like priming, mesendoderm specification, and cardiac progenitor induction, followed by their expansion, terminal differentiation, and maturation (Kempf et al., 2016). Previously, a cardiac differentiation protocol to modulate the WNT signaling pathway in a heart development-like fashion using small molecules was reported; this included early upregulation of the WNT pathway for primitive streak-like mesendoderm priming followed by latter suppression for cardiac progeny specification (Lian et al., 2012). The glycogen synthase kinase 3 (GSK3β) inhibitor CHIR99021 (CHIR) was used to activate the WNT pathway, which inhibits the destruction complex of β-catenin and results in its accumulation. The differentiation outcome is therefore strongly dependent on the β-catenin concentration, which is sensitive to CHIR concentration, the timing of CHIR supplementation, and the timing of subsequent WNT pathway suppression by chemical factors such as IWP2, IWR1, or Wnt-C59 (Lian et al., 2012). Downstream of the chemical WNT pathway modulation, other autocrine and paracrine pathways are activated, in particular, TGF and NODAL, which occur in a cell density-dependent manner previously termed the bulk cell density (BCD; Kempf et al., 2016). Therefore, the process outcome is also influenced by the inoculation and proliferation-dependent BCD, particularly during the first 24 h of differentiation induction, which ultimately impacts the CM yield and content. Even in tightly controlled systems, the inherent complexity of these differentiation steps and the high number of molecular, cellular, environmental and physical parameters makes it challenging to consistently obtain uniform results, which is highly desirable for industrial and clinical applications. Notably, in reply to WNT pathway modulation, differentiation can result not only in the formation of CMs but also in multiple non-CM lineages of endodermal and/or mesodermal origin including, for example, endothelial cells (ECs) and fibroblasts (FBs) (Kempf and Zweigerdt, 2018). Moreover, hPSC-derived CMs may represent a subtype-specific mixture, including cardiac pacemaker-, atrial- and ventricular-like phenotypes, as suggested by their electrophysiological features (Zhang et al., 2009).

Establishing robust and scalable CM production processes from hPSCs is critical for obtaining clinically relevant cell numbers. In contrast to conventional cell culture in a dish, instrumented STBRs have the advantage of enabling continuous monitoring of numerous process parameters. For example, online measurements of pH and dissolved oxygen (DO) provide uninterrupted information on the cellular environment. Furthermore, bioreactor-based suspension culture enables continuous collection of process samples in adequate quantities for offline monitoring of additional parameters such as time-resolved changes in the aggregate size, cell-density (growth kinetics), and glucose and lactate levels, all of which provide valuable information on cell viability, proliferation, differentiation, and their metabolic status. The cultivation of hPSCs as cell-only aggregates in STBRs enabled the production of millions of cells within one batch (Kropp et al., 2016). A scalable method utilizing spinner flasks for differentiating high purity CMs from hPSCs with scales up to 1 L has been reported with CM content of >96% (Chen et al., 2015). In that study, the impact of several parameters such as small molecule concentration, aggregate size, agitation rate, glucose and lactate concentrations, DO concentration, pH, and induction timing on cardiac differentiation was evaluated. Furthermore, STBR-based suspension culture in relatively large scales (100 mL up to 1 L) has been carried out for the production of CMs from hPSC aggregates (Kempf et al., 2014; Chen et al., 2015; Fonoudi et al., 2015; Halloin et al., 2019). In all these studies, successful CM induction was reported typically yielding >85% CM content. However, it was also highlighted that large inter-process variability exists, which may lead to >96% CM content in some processes but <60% CM content in independent process repeats. In the context of this study, a yield of >90% CM content is considered *sufficient*, i.e., a process success, whereas <90% CM content is considered *insufficient*, i.e., process failure. Given the above indicated multifactorial complexity along the transition of hPSCs into contractile CMs, it is currently not apparent which individual parameter(s) or their combinations are directly involved in causing the undesired process heterogeneity. This fact is a key challenge for the future envisioned CM production at GMP-compliant, industry compatible conditions in multi-liter scales.

Machine learning techniques have been used in bioprocess development for the identification of critical experimental factors, for example, to aid in the optimization of the production of several proteins and cell lines. Du et al. (2016) presented a method for identifying essential model parameters of computer models of cardiac sodium channels using Gaussian process modeling, and for reducing the complexity of the models. Charaniya et al. (2010) identified several process parameters with strong associations to outcomes for the manufacturing of recombinant proteins using support vector machines. Caschera et al. (2011) successfully increased the yield of their cell-free protein synthesis process by 350% via designing experimental conditions using artificial neural networks (ANNs), which were recently also applied to find the optimal harvest time for xylitol production by Pappu and Gummadi (2016). Others have looked at maximizing protein production by identifying and optimizing key factors in the fermentation process, also using ANNs (Sinha et al., 2014; Amiri et al., 2015).

Metabolic pathways are another target for manipulation to maximize protein production. The pathways have been modeled using both principal component analysis (PCA) (Alonso-Gutierrez et al., 2015) and an ensemble of ANNs (Zhou et al., 2018). Sokolov et al. (2017) used regression techniques to achieve improved monoclonal antibody quality, which was measured with 14 quality attributes, including the quantities of charge variants, aggregates, and glycoforms. These attribute values were optimized by changing experimental conditions such as the cell culture media formulations and conditions (pH, temperature) using PCA and partial least squares regression models. Kotidis et al. (2019) determined ranges of process inputs that would consistently meet several protein product quality indicators using global sensitivity analysis.

Although cardiac differentiation from hPSCs in suspension culture has recently become more efficient and robust (Halloin et al., 2019), there still exist opportunities for further understanding and improvement of these processes. For example, limited knowledge exists on how perturbations in bioreactor parameters and culture conditions affect cell yield and CM content. Utilizing data-driven modeling and machine learning techniques to understand mesendoderm differentiation, in particular cardiac priming, is an advantageous initial model. Notably, cardiac differentiation is a somewhat easier and better-studied model of lineage differentiation (Matsa et al., 2014; Kempf and Zweigerdt, 2018; Mummery, 2018) compared to more complex cell types such as hematopoietic lineages (Ackermann et al., 2018). Moreover, the *in vitro* cardiac differentiation process can be controlled by a low number of chemical factors such as the WNT modulators CHIR and IWP and can be completed in 10–14 days from hPSC seeding. Furthermore, there is substantial knowledge and existing data for hPSC-CM differentiation in STBRs due to the large interest in this cell type, including the first mathematical model to understand the controlling factors for cardiac mesoderm specification (Gaspari et al., 2018).

**FIGURE 1:**
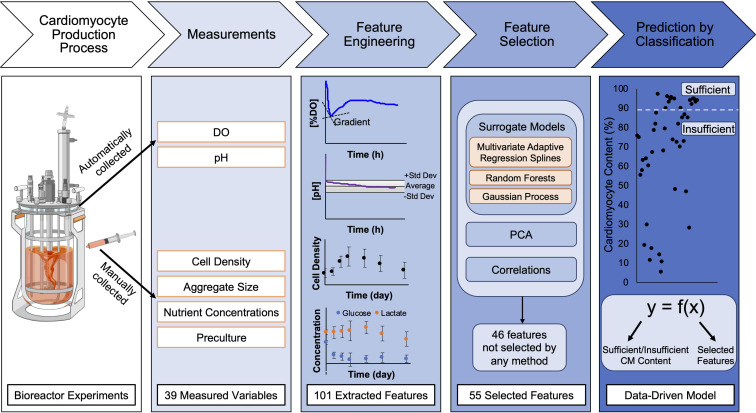
Schematic of process to generate data-driven models for prediction of final CM content from bioreactor experiments. Human pluripotent stem cells (hPSCs) were seeded in bioreactors for expansion for 48 h prior to initiation of cardiac differentiation. To initiate cardiac differentiation, a WNT amplifier, CHIR, was added for 24 h followed by 48 h of WNT inhibition using IWP2. Differentiation experiments lasted 10 days and endpoint analysis for final CM content in bioreactors was performed on differentiation day 10. During the time course of differentiation, the dissolved oxygen (DO) and pH were continually monitored. Samples were collected from the bioreactor during differentiation to analyze cell density, aggregate size, nutrient concentrations, and preculture conditions. From the 39 measured variables throughout differentiation, feature engineering was performed to extract 101 features; for example, the continuous data was separated by differentiation day to obtain averages, gradients, and second derivatives for each day. Feature selection was performed using surrogate models, principal component analysis, and correlations to determine which of the extracted features impacted the variance in the data and outcome of differentiation. After feature selection was performed, data-driven models were developed using surrogate models (MARS, RF, and GP) to predict final CM content by classification of the data in two categories (created with Biorender.com).

Based on our recent experience in a STBR-based hPSC-CM differentiation process (Halloin et al., 2019), we have here defined the induction of 90% CM content or higher as a “process success”; in contrast, induction of less than a 90% CM content is defined *insufficient* or a “process failure.” Using machine learning techniques like classification for the interpretation of existing experimental data sets, the goal of this paper was to identify the most informative parameters predictive of the CM differentiation efficiency in a bioreactor platform. As a result, we here report predictive parameters and algorithms for this process (Figure 1). The study supports both the early interruption of failing processes (providing cost and time savings) and the rationale for further process modifications that may ultimately avoid future process failures.

## Materials and Methods

### Basic hiPSC Culture and Directed Differentiation in a Stirred Tank Bioreactor System

The hiPSC line Phoenix (Haase et al., 2017) was cultured in E8 medium as described (Kempf et al., 2015; Halloin et al., 2019). In brief, cells were seeded at 0.5 × 10^4^ cells/ml on Geltrex-coated cell culture flasks in E8 medium supplemented with 10 μM Y-27632 and passaged every 3.5 days.

**FIGURE 2:**
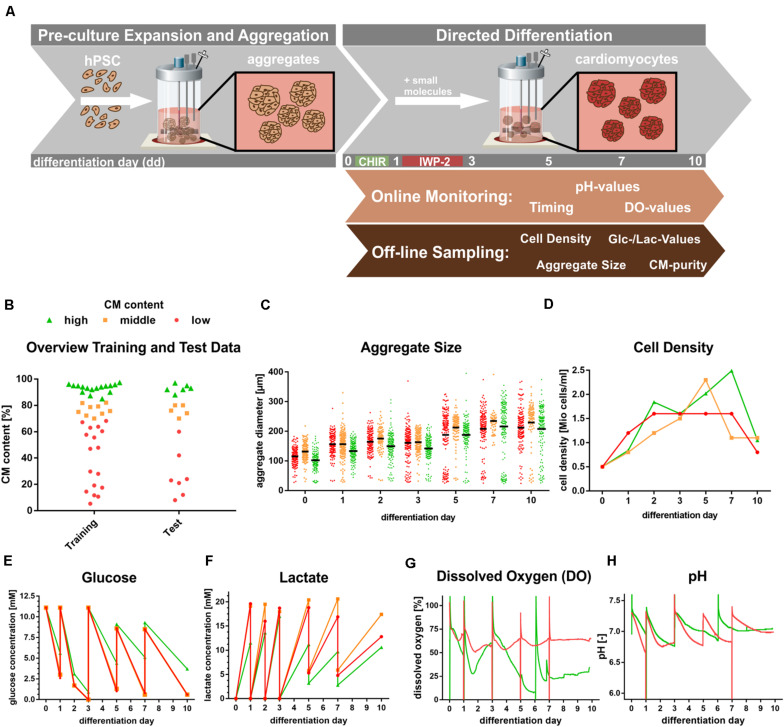
Generation of experimental data. **(A)** Schematic bioreactor set-up of hPSCs expansion in suspension culture followed by directed cardiac differentiation by chemical WNT pathway modulation over 10 days. **(B)** Overview of Training and Test data spreads based on flow cytometry analysis of CM-specific MHC-expression. Definition: Endpoint analysis of processes resulting High ≥85% (green), middle 70–85% CM (orange) and low ≤70% CM content (red). **(C–H)** Representative patterns of process parameters, exemplifying processes typical for high (green), middle (orange), and low (red) CM content at process endpoint (day 10). **(C)** Aggregate size distribution at respective days of differentiation. **(D)** Representative cell density kinetics. **(E,F)** Glucose and lactate values over the course of differentiation. **(G,H)** Representative dissolved oxygen and pH patterns monitored via online analysis over the course of differentiation. Please note that the orange Glucose pattern in **(E)** is hardly visible due to the close overlay of the red pattern. In **(G,H)** only representative DO and pH patterns for respective high (green) and low (red) CM content processes are displayed to avoid loss of clarity by overlapping patterns.

For process “pre-culture expansion and aggregate formation” (Figure 2A), a STBR system (DASbox, Eppendorf) was inoculated with 5 × 10^5^ hiPSCs/ml in E8 supplemented with 10 μM Y-27632 at a final volume of 150 ml per reactor vessel. Approximately 24 h after inoculation, perfusion was initiated with 4.2 ml/h fresh medium, as described in Kropp et al. (2016). For inducing chemically defined, directed differentiation, the cell density of the pre-culture was adjusted at 48 h after single cell inoculation to achieve 5 × 10^5^ cells/ml in differentiation medium CDM3 with 5 μM CHIR and 5 μM Y-27632. After 24 h (dd1) the medium was replaced by CDM3 (Burridge et al., 2014) with 5 μM IWP-2; at 72 h (dd3; and every 2–3 days thereafter) 100 ml consumed medium was collected from the bioreactor and replaced with fresh CDM3 (Halloin et al., 2019).

### Data Collection, Cell Sampling, Analysis, and Process Grouping Based on CMs Content

Over the course of the cultivation/differentiation process, data was collected as schematically shown in Figure 2. DO and pH were constantly measured online, whereas data on the cell density, aggregate diameter, nutrient concentration and CM content were evaluated offline. Bioreactor-derived sampling of cell aggregates in 2 ml medium was performed as previously described (Kempf et al., 2014; Kropp et al., 2016; Halloin et al., 2019). For aggregate analysis, microscopic images were taken (Axiovert A1; Zeiss); on these images, a minimum of 100 aggregates for each sample were assessed by an ImageJ macro to automatically define the mean diameter.

For cell density assessment and flow cytometry analysis, aggregates were dissociated and automatically counted (Vi-CELL XR; Beckman Coulter); in the remaining supernatant, glucose and lactate concentration was measured (BIOSEN C-line; EKF Diagnostic). For flow cytometry, 2.0 × 10^5^ cells were fixed, permeabilized and incubated with the following CMs-specific primary antibodies: anti-cardiac Troponin T (1:200, clone 13-11, Thermo Scientific), anti-sarcomeric α-actinin (1:800, EA53, Sigma-Aldrich or 1:20, REA402, Miltenyi Biotec), anti-myosin heavy chain (1:20, MF20, Hybridoma Bank); after incubation with appropriate Cy5-conjugated antibodies (1:200, Jackson ImmunoResearch) data were acquired on an Accuri C6 flow cytometer (BD Biosciences) or MACSQuant Analyzer 10 (Miltenyi Biotec) and analyzed using FlowJo software (Flowjo, LLC).

By flow cytometry analysis of dd10 derived samples using the 3x CMs-specific antibodies outlined above, the average CMs content for each differentiation process was assessed; processes were consequently grouped into those with either *sufficient* or *insufficient* CM content, with *insufficient* content being defined as a process with a CM content of <90% (Figure 2B).

## Computational Methods and Theory

### Employing Experimental Data for Feature Engineering

Experimental data used in computational analysis and model development were collected from 58 cardiac differentiation processes in bioreactors; notably, all processes performed in the relevant experimental setup were included in the study without any type of pre-selection procedure to explicitly exclude any investigator-dependent bias. Each of the differentiation process represents a single experimental datapoint to be used for model construction. In a first step, data sets from 42 of these processes were randomly chosen and used for constructing predictive models, while data sets from the remaining 16 processes were reserved for testing the models’ performance.

From the data, a set of potential input variables, which we refer to as “bioprocess features,” for use in predictive models was generated with the goal of this set fully describing the experimental conditions over the entire differentiation process. For model construction using machine learning, a feature is an individual measurable or derived properties (using measured) of the system that is being modeled. Available experimental conditions included the rotation speed in the bioreactor and measurements such as differentiation day (dd) dependent cell densities, aggregate sizes, and nutrient concentrations, and measurements of DO concentration and pH over the course of the experiment. Examples of bioprocess features and how the data was collected are summarized in Figures 1, 2.

**FIGURE 3:**
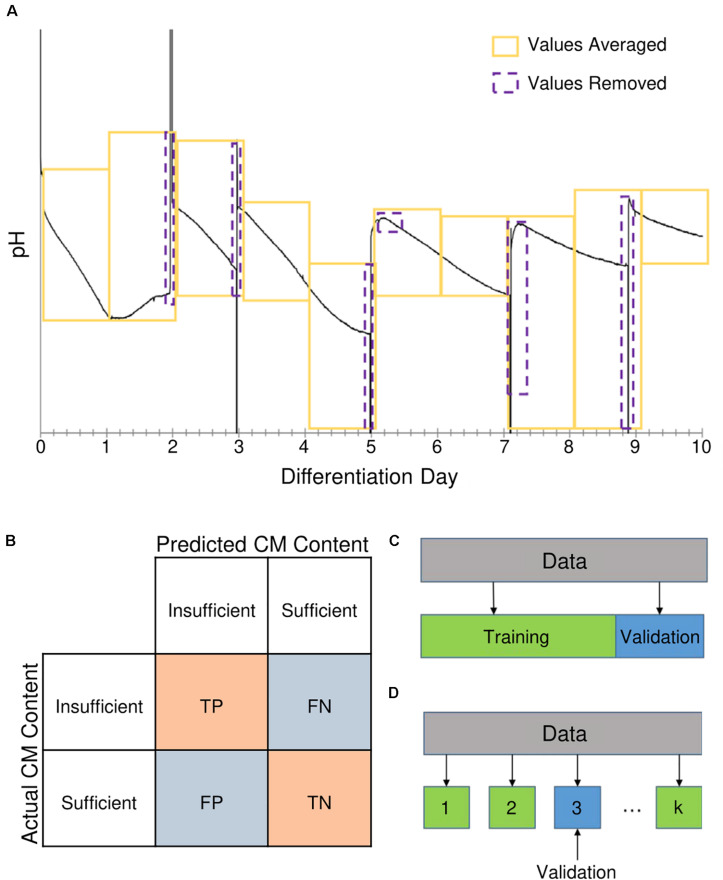
Methods used for generation of features and evaluation of model performance. **(A)** Continuous measurements for pH and dissolved oxygen (DO) concentration were averaged by differentiation day. Yellow boxes represent time periods measurements were averaged over (i.e., differentiation days). Purple boxes represent measurements taken during media changes that were removed from the averaging. **(B)** Model performance was evaluated based on metrics from the classification confusion matrix. When the model correctly identified the result as “insufficient” or “sufficient,” it was labeled as a true positive (TP) or true negative (TN) result, respectively. A “sufficient” result incorrectly identified as “insufficient” was considered a false positive (FP), whereas an “insufficient” result incorrectly identified as “sufficient” was a false negative (FN). **(C)** During model training, the performance was validated using leave one out (LOO) cross-validation and Monte Carlo (MC) cross-validation. In LOO cross-validation, a single data point was set aside while the model was built with the remaining data points; a prediction is then obtained for the data point that was left out. This process was repeated for each data point, resulting in a prediction for each data point that were used in calculating performance metrics. **(D)** For MC cross-validation, a set of data was randomly selected to be excluded for validation, and the model was built using the remaining data.

The DO concentration and pH measurements were included as features by averaging their values over each day of the differentiation, illustrated in Figure 3A. Additional features were engineered from this data, as well as other time-dependent measurements, to capture how the conditions in the bioreactor were changing over time. These additional features were generated by estimating gradients and second derivatives for the cell density, aggregate size, DO concentration, and pH measurements, resulting in a final set of 101 potential bioprocess features. The full list of bioprocess features is provided in Supplementary Table 1. Gradients and second derivatives were estimated using Eqs. (1) and (2),

(1) $$g_{t_{i}}=\frac{y_{t_{i}}-y_{t_{\text{i}-1}}}{t_{i}-t_{\text{i}-1}}$$

(2) $$h_{t_{i}}=\frac{g_{t_{i}}-g_{t_{\text{i}-1}}}{t_{i}-t_{\text{i}-1}}$$

where *g*_*t*_*i*__ and *h*_*t*_*i*__ are the gradient and second derivative, respectively, of bioreactor condition *y* at timepoint *t*_i_.

### Feature Selection Methods

Because of the large number of features (101) compared to the number of experimental data points (58), feature selection was performed on the available data to discover which of the bioprocess features were most influential and predictive of the cardiomyocyte content on dd10. The feature selection methods employed include correlation coefficients, PCA, and the built-in feature selection capabilities of the machine learning techniques investigated for predictive modeling. Two sets of features, Feature Set 1 and Feature Set 2, were considered, each with bioprocess features measured at earlier points in the differentiation process. Feature Set 1 consists of all the collected bioprocess features measured up until the seventh differentiation day 7 (dd7). Feature Set 2 consists of bioprocess features measured up until the fifth differentiation day 5 (dd5).

The dd7 and dd5 timepoints were chosen in order to use as much data as possible without using any data near the endpoint of the differentiation, such as dd9. Preliminary proof-of-concept studies revealed that classification was possible using data collected up to and including dd7, initiating analysis investigating the possibility of earlier predictions. Based on this analysis, dd5 was chosen as the earliest possible timepoint, as classification using data from earlier points in the differentiation did not yield satisfactory predictive capabilities.

#### Correlations

##### Pearson correlation coefficient

The Pearson correlation coefficient measures the linear relationship between two variables. Its value ranges from −1 to 1. A value of −1 corresponds to a perfect negative linear relationship between the variables, while a value of 1 indicates a positive linear relationship. A value of 0 demonstrates no linear correlation between the variables (Soper et al., 1915).

##### Spearman correlation coefficient

The Spearman correlation coefficient measures the strength and direction of a monotonic relationship between two variables. Its value ranges from −1 to 1. A value of 0 indicates no correlation between the variables. Values of −1 or 1 indicate a perfect negative or positive correlation, respectively (Spearman, 1904).

#### Principal Component Analysis (PCA)

Principal component analysis is a statistical dimension reduction tool. The method transforms a set of possibly correlated variables to uncorrelated principal components (PCs). It identifies a new set of orthogonal axes in the direction of the highest variance of the data. Each of the axes, which is a linear combination of original axes, represents a PC. Principal components are assigned in ordinal format with the first PC explaining the highest percentage of the variance and the last PC the least. The PCs with the lower ranks are generally not considered in further analysis reducing the number of dimensions while preserving much of the original variance (Hotelling, 1933).

### Machine Learning Techniques

#### Multivariate Adaptive Regression Splines

Multivariate adaptive regression splines (MARS) models are non-parametric statistical models that consist of a linear summation of basis functions (Friedman, 1991). In general, basis functions are either a constant, a hinge function, or the product of two or more hinge functions. For the MARS models trained in this study, the Sci-Kit Learn pyEarth software package was used (Pedregosa et al., 2011). Detailed information MARS models and the other machine learning techniques described in this section are provided in the Supplementary Material (see section “Extended Machine Learning Technique Descriptions”).

#### Random Forests

Random forests (RFs) are a machine learning method that utilizes a set of decision trees for predicting an output based on input data. Each tree is built independently based on a random subspace of the training data. The final output of a random forest model is determined by averaging the output value of every tree in the forest (Breiman, 2001). The features are selected according to the importance level calculated by the random forest model. The importance level is based on the impact of a feature on improving the separation of the data in each decision node of the tree. For the RF models trained in this study, the Sci-Kit Learn RandomForestRegressor software package was used to train forests with 5 trees (Pedregosa et al., 2011).

#### Gaussian Process Regression

Gaussian process regression (GPR) is a non-parametric machine learning method where the prediction of the output corresponding to an unknown input is calculated based on a weighted average of outputs for known inputs using a similarity metric: the kernel function (Rasmussen and Williams, 2005). The kernel function used for all GPR models in this paper is a radial basis function.

Gaussian process regression can be used for feature selection with its built-in automatic relevance determination (ARD) method. Further sensitivity analysis (Eq. 4) on the ARD results (Blix and Eltoft, 2018) provides an even greater separation of the features for selection. For the GPR models trained in this study, the Sci-Kit Learn GaussianProcessRegressor software package was used (Pedregosa et al., 2011).

### Cardiomyocyte Content Classification

A binary process classification based on the CM content (%) at process endpoint (dd10) was applied, and the two classes defined were: “*sufficient*” for: CM content equal to and above 90%, and “*insufficient*” for CM content below 90%. A binary classification model was chosen after an initial analysis with a multiclass model revealed that the available bioprocess data was not rich enough to train a multiclass model at this time.

To enable CM content prediction based on early process data, two regression models using MARS and GPR were built, and the data points were assigned to their classes (i.e., *sufficient* or *insufficient*) based on this predicted CM content value. For RFs, the classification is conducted directly using the classifier models, constructed by the RF.

To evaluate and compare the performances of the classification models, four metrics were considered: accuracy, precision, recall, and the Matthews correlation coefficient (MCC). The range for the first three metrics is zero to one, and MCC is between −1 and 1. These metrics are calculated based on the confusion matrix (Sokolova and Lapalme, 2009), which is illustrated in Figure 3B. The confusion matrix describes the performance of a classification model (algorithm). In this paper, we assign the *insufficient* CM content class as the positive class (Figure 3B) and the *sufficient* class as the negative one (Figure 3B). The error of the predictions is broken down for each class using the confusion matrix. The four cells of the confusion matrix correspond to true positive, false negative, false positive, and true negative. The values associated with each of the components give information about how many of the positive/negative classification results were correctly predicted by the model.

#### Classification Model Performance Metrics

##### Accuracy

Accuracy calculates the proportion of correct classifications (Sokolova and Lapalme, 2009). According to Eq. (5), accuracy is the number of all true positives and negatives compared to all prediction results. Accuracy of one indicates that the classification has been conducted accurately and that all the points with *sufficient* or *insufficient* CM content have been included in the right class (*FP* + *FN* = 0). Zero accuracy defines a totally wrong classification model, which is not able to predict the label of the points correctly.

(5) $$A\,c\,c\,u\,r\,a\,c\,y=\frac{\left(T\,P+T\,N\right)}{\left(T\,P+T\,N+F\,P+F\,N\right)}$$

##### Precision

Precision (Eq. 6) gives information about the proportion of the times the points identified as positive were truly positive (Sokolova and Lapalme, 2009). Precision of one means that all the positive results are actually positive outcomes. When a classifier model with precision of one predicts *insufficient* CM content for a point, it is supposed to have *insufficient* CM content in practice. Value of zero for precision indicates that all the identified positive outcomes are false.

(6) $$P\,r\,e\,c\,i\,s\,i\,o\,n=\frac{T\,P}{\left(T\,P+F\,P\right)}$$

##### Recall

Recall, Eq. (7), is the proportion of actual positive results which were identified as positives (Sokolova and Lapalme, 2009). The value of one for recall demonstrated that the model is able to classify all the actual positive results as positive. In CM content case, all the *insufficient* points would be identified as *insufficient* using a model with recall equal to one. When all the positive classes are falsely identified negative, the value of recall equals to zero.

(7) $$R\,e\,c\,a\,l\,l=\frac{T\,P}{\left(T\,P+F\,N\right)}$$

##### Matthews’s correlation coefficient (MCC)

Matthews’s correlation coefficient (Eq. 8) defines the correlation between the predicted and actual classifications for all data points (Matthews, 1975). Value of one for MCC means there is a strong correlation between the predicted results and the actual values, indicating that the predicted label is correct for all the points. Value of −1 for MCC metric demonstrates a strong inverse correlation. Values of zero for MCC corresponds to no correlation between the predicted and actual results.

(8) $$M\,C\,C=\frac{\left(T\,P\times T\,N\right)-\left(F\,P\times F\,N\right)}{\sqrt{\left(T\,P+F\,P\right)\,\left(T\,P+F\,N\right)\,\left(T\,N+F\,P\right)\,\left(T\,N+F\,N\right)}}$$

The classification model performance metrics, accuracy, precision, recall, and MCC, were calculated using two different cross-validation techniques: (1) leave one out (LOO) cross-validation (Figure 3C), and (2) Monte Carlo (MC) cross-validation (Figure 3D). Cross-validation is a tool for assessing how well a model can be generalized to new data, which the model has never seen. In MC cross-validation, a set of data is selected randomly to be excluded for validation, and this data set is called the validation set. The model is built using the remaining data, and the model is used to predict the classes for the validation set (Burman, 1989). These predictions are used to calculate performance metrics. In LOO cross-validation, a single data point is set aside (i.e., left out) for validation. The model is built using the remaining data points, and a prediction is obtained for the data point that was left out. This process is repeated for each data point, resulting in a prediction for each. Figures 3C,D illustrates how these cross-validation methods were used for evaluating the performance metrics (Wong, 2015).

## Results and Discussion

### Feature Selection

**FIGURE 4:**
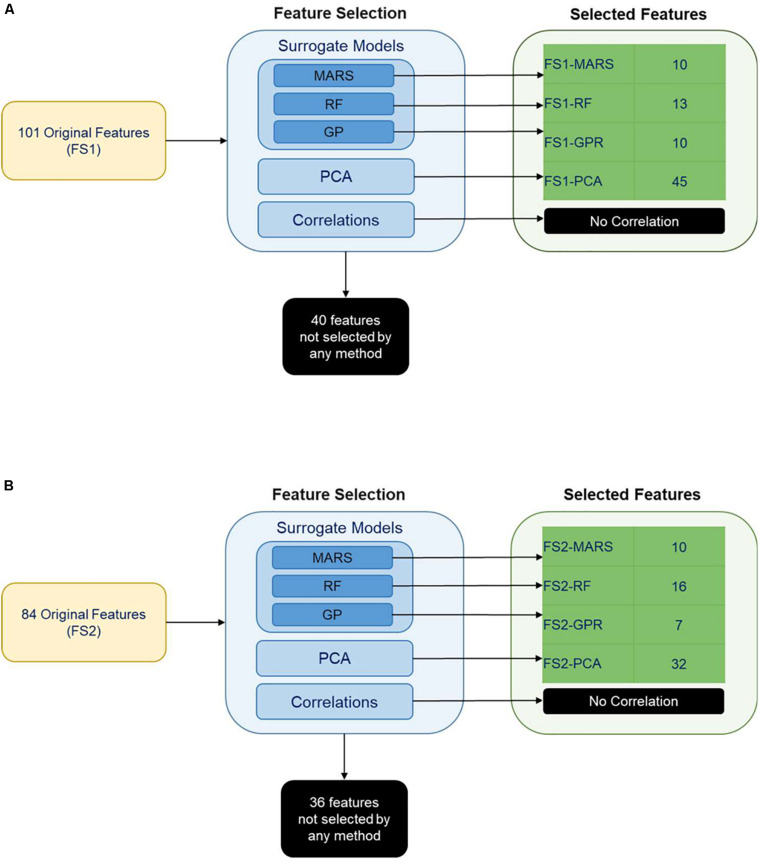
Feature selection results for features selected from **(A)** feature set 1 and **(B)** feature set 2. MARS (Multi-adaptive regression splines), RF (Random Forest), GPR (Gaussian process regression) were the surrogate models implemented for the selection of the features that have a significant impact on classification. The other feature selection methods were PCA (Principal component analysis) and correlations between the variables.

Two different sets of features were considered for building the classification models. Feature Set 1 contained all potential bioprocess features measured through dd7. Feature Set 2 contained all features measured through dd5. Feature selection was performed on each feature set separately to identify potential features for predicting CM content class on dd10. Classification models were then built using these potential feature sets. We employed PCA, and built-in capabilities of MARS, RFs, and GPR for feature selection. Feature selection resulted in eight potential feature sets for classifying the CM content on dd10. A visual summary of the feature selection results is provided in Figure 4.

Principal component analysis yielded five principal components (FS1-PCA) that explained 94% of the variance in the input data for Feature Set 1 (Figure 4A) and yielded four principal components (FS2-PCA) that explained 94% of the variance for Feature Set 2 (Figure 4B). None of the principal components or bioprocess features strongly correlated with the CM content. The strongest linear correlation between a feature and the CM content was −0.51, and that feature was the time that the differentiation media was supplemented with WNT inhibitor IWP2. This lack of correlation indicates that none of the individual bioprocess features alone suffices to make a prediction on the CM content and that other means, such as machine learning techniques, are necessary to investigate the relationship. The number of features selected by each machine learning technique is provided in Figure 4. A list of features selected by each method is available in Supplementary Tables 1, 2. Tables 1, 2 give a listing of the bioprocess features selected by each modeling technique for Feature Sets 1 and 2, respectively.

**TABLE 1:** Bioprocess features selected by each surrogate modeling technique from Feature Set 1 (X = selected).

Feature	FS1-RF	FS1-GPR	FS1-MARS
Average DO concentration dd0	X		
Average DO concentration gradient d0	X		X
Average DO concentration gradient dd2		X	
Average DO concentration gradient dd6	X		
Average DO concentration gradient dd7		X	
d0 average pH gradient	X		
dd0 average pH gradient		X	
dd0 average acceleration of cell density normalized DO gradient	X		
dd0–dd1 cell density gradient		X	X
dd1 aggregate size			X
dd1 cell density	X		
dd2 average pH	X		
dd7 average pH		X	
dd3 aggregate size			X
dd3 average acceleration of DO gradient			X
dd3 average pH gradient	X		
dd3 average acceleration of cell density normalized DO gradient			X
dd5 average pH gradient			
dd5–dd7 aggregate size gradient	X		
dd5–dd7 cell density gradient			X
dd7 cell density	X	X	
Cell density normalized DO concentration dd2			X
Cell density normalized DO concentration dd3		X	X
Cell density normalized DO concentration dd7	X		
Average cell density normalized DO concentration gradient dd2	X	X	
Average cell density normalized DO concentration gradient dd5		X	
Average cell density normalized DO concentration gradient dd7	X		
IWP2 treatment time [h]			X
Preculture time [h]		X	

**TABLE 2:** Bioprocess features selected by each surrogate modeling technique from Feature Set 2 (X = selected).

Feature	FS2-RF	FS2-GPR	FS2-MARS
Average DO concentration d0	X		
Average DO concentration dd0	X		
Average DO concentration dd2			X
Average DO concentration dd4	X		
Average DO concentration gradient d0	X		
Average DO concentration gradient dd2	X	X	
Average DO concentration gradient dd4	X		X
Average DO concentration gradient dd5	X		
d1 average pH gradient			X
dd0 aggregate size			X
dd0 cell density			X
dd0–dd1 cell density gradient		X	
dd1 average pH	X		X
dd1 average acceleration of cell density normalized DO gradient	X		
dd2 aggregate size	X		
dd2 average acceleration of DO gradient		X	
dd2 average pH			X
dd2 average pH gradient	X		
dd2 average acceleration of cell density normalized DO gradient	X		
dd2–dd3 aggregate size gradient	X		
dd3–dd5 cell density gradient			X
dd4 average pH gradient		X	
dd5 average acceleration of cell density normalized DO gradient	X	X	
Cell density normalized DO concentration dd1	X		
Average cell density normalized DO concentration gradient dd5		X	
IWP2 treatment time [h]		X	X
Overall aggregate size gradient	X		
Overall density gradient			X

The features selected by MARS, RF, and GPR encompassed features with known biological implications as well as potential mechanistic explanations, although the impacts of the features in combination have not been previously investigated. It is well established in developmental biology, that a bi-phasic WNT pathway modulation is essential for cardiac mesoderm specification and subsequent CM formation. As outlined in Figure 2A, cardiac differentiation in this study was controlled by chemical WNT modulators added in a temporal pattern. hPSC differentiation was initiated by the addition of the chemical WNT pathway accelerator CHIR on dd0 for 24 h followed by 48 h of WNT attenuation through addition of IWP2. The small molecule, CHIR, is solely sufficient to induce early primitive streak (PS)-like priming in hPSCs in a concentration-dependent manner (Lian et al., 2012; Kempf et al., 2016; Gaspari et al., 2018). The higher the CHIR dose, the faster hPSC differentiation will progress from an (anterior) endoderm-like fate toward cardiac mesoderm specification and subsequently toward more posterior fates such as somatic mesoderm-like PS priming. The dynamics of the CHIR induced differentiation processes, particularly during the first 24 h of differentiation, play an important role regarding the degree of heterogeneity of the cardiac differentiation process, of which identifying the underlying factors is one of the objectives of this study. However, our recent work revealed that the accelerating effect of CHIR on differentiation progression is counteracted by cell-secreted factors such as the transforming growth factor beta (TGFb) family members, Nodal signaling antagonists left-right determination factor 1 (LEFTY1) and CERBERUS (CER1) (Kempf et al., 2016). The cell density directly impacts the accumulation of such secreted factors, particularly during the first 24 h of differentiation (dd0–dd1) and plays a key role in the subsequent specification of cell fates and subsequently the CM content (assessed on dd10 in this study); this suggests that the selected features “*dd0–dd1 cell density gradient*” and “*dd1 cell density*” identified by our modeling approach, have known mechanistic impacts on CM differentiation.

It is of biological relevance that the “*IWP2 treatment time*” was also selected as an important feature for model development, given the importance of temporal WNT signaling for proper lineage differentiation in embryogenesis and *in vitro* as well (Ueno et al., 2007; Burridge et al., 2014; Halloin et al., 2019). However, the feature selections “*dd1 average acceleration of DO gradient*” and “*dd3 cell density normalized DO concentration*” are non-obvious, interesting new observations. These model-extracted features strongly suggest that an acceleration/deceleration of the (cell density-normalized) DO gradient at a specific process interval impacts the final CM content. This may relate to process-dependent changes in the overall density of (oxygen-consuming) cells, as well as differentiation stage-dependent changes in cells’ metabolism. During the differentiation process, the metabolism switches from predominantly glycolysis typical of undifferentiated hPSCs (Kropp et al., 2016), toward oxidative phosphorylation as a result of CM specification and maturation (Hu et al., 2018). Thus, our modeling approach suggests a concrete novel hypothesis requiring future experimental validation. Example values for these DO related features are illustrated in Supplementary Figure 1.

After mesoderm priming and cardiac progeny specification during the first ∼72–96 h of our differentiation protocol, progressive differentiation into functional, sarcomere protein expressing CMs occurs in the period between dd5 and dd10 (Halloin et al., 2019). It is thus interesting to note the MARS model selection of the features “*dd5–dd7 cell density gradient*” and “*dd5 average DO concentration gradient*” as being important for the CM content. A possible interpretation of this result is that the dd5–dd7 cell density/DO patterns may impact (i.e., inhibit or promote) the maturation of cardiac progenies into functional CMs. The cell density-dependent secretion of factors during this differentiation stage may impact the sarcomere protein expression including isotype switches resulting in CM maturation (Hu et al., 2018) equivalent to cell density-dependent mechanisms of primitive streak-like priming during the first 24 h of differentiation. Furthermore, since the DO gradient correlates to the cell density gradient, the “*dd5 average DO concentration gradient*” feature may relate to the “*dd5–dd7 cell density gradient*” feature.

Using the feature selection components of these data-driven models provides the possibility of examining bioprocess features in combination and in more detail. Some of the identified features presumably have known mechanistic impacts on the outcome of cardiac differentiation, as outlined above, thereby indicating the validity of our approach. However, there are several features identified by the feature selection but have not been previously examined or identified as important for predicting the CM content, including the dd5–dd7 cell density and DO gradients.

On the other hand, it is worth noting that, for example, supraphysiological glucose concentration has been found to impact the cardiac differentiation of hESC (Crespo et al., 2010; Yang et al., 2016). In our model, the glucose and the related lactate concentration patterns were not classified as important features, i.e., were not identified as being predictive of the CM content in our differentiation process. But this finding is not excluding, *per se*, that the glucose concentration is important for the cardiomyogenesis of hPSCs.

This in mind, it is important to note, that the impact of a selected feature on the differentiation outcome does not presumptively indicate a mechanistic relationship. This impact may be correlative only (rather than causative), potentially a result of processes not captured by the input data or through feature engineering. However, the identified features are informative for guiding future validation experiments; these can then be used to build more mechanistic models for understanding and potentially reducing variability in cardiac differentiation.

### Classification Results

**FIGURE 5:**
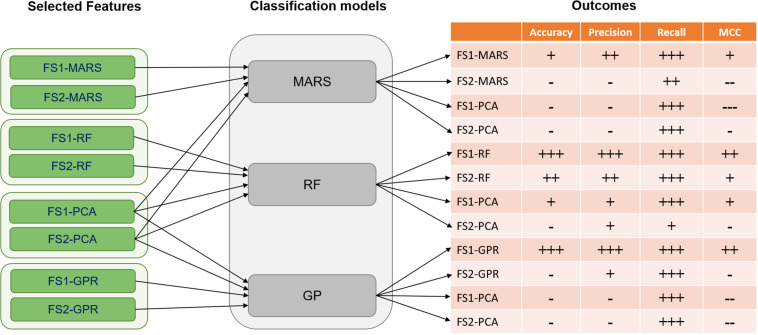
Classification results were based on four metrics including accuracy, precision, recall, and MCC (Matthews correlation coefficient) for selected features of FS1 and FS2. The classification models of MARS (Multi-adaptive regression splines), RF (Random Forest), and GPR (Gaussian process regression) were implemented. For accuracy, precision, and recall the values are categorized as follows: (—) for values < 0.3, (–) for 0.3 ≤ values < 0.6, (-) for 0.6 ≤ values < 0.7, (+) for 0.7 ≤ values < 0.8, (++) for 0.8 ≤ values < 0.9, and (+++) for value ≥ 0.9. Moreover, for the MCC metric, the categorization was done as: (—) for values < 0, (–) for 0 ≤ values < 0.1, (-) for 0.1 ≤ values < 0.3, (+) for 0.3 ≤ values < 0.7, (++) for 0.7 ≤ values < 0.9, and (+++) for value ≥ 0.9.

Classification models were constructed for predicting the outcome of the bioreactor experiments on dd10 using features measured up to dd7 and up to dd5, using each of the machine learning techniques described in Sections “Multivariate Adaptive Regression Splines,” “Random Forests,” and “Gaussian Process Regression.” The models were built using the bioprocess features selected from Feature Set 1 (for predicting using features measured until dd7) and Feature Set 2 (for predicting using features measured until dd5). Results for classification model performance for each of the eight feature sets from the feature selection are summarized in Tables 3, 4. Results were obtained using LOO cross-validation and are presented for both the bioprocess features selected by the built-in feature selection for each model, as well as for the PCs obtained from PCA. Both feature sets contained 42 data points chosen from the original set of 58 experiments for training. A visual summary of the results for each classification method with its associated feature sets is depicted in Figure 5. For all of the feature sets generated from Feature Set 1, for all of the techniques investigated, classification using the model-selected features always had a better performance than the principal components from PCA. Only two classification model-feature set combinations achieved favorable results for all four of the performance metrics, which is illustrated in Figure 5. RFs trained with feature set FS1-RF and GPR trained with FS1-GPR perform similarly for predicting if CM content will be *insufficient* for continuing the experiment. Both methods obtained accuracies of 90% and precisions around 90%, meaning that if a model predicts the CM content will be *insufficient*, there is a 90% probability that it is *insufficient*.

**TABLE 3:** Classification model performance (calculated with LOO cross-validation) for models trained with features from Feature Set 1.

	MARS		RFs		GPR	
	FS1-MARS	FS1-PCA	FS1-RF	FS1-PCA	FS1-GPR	FS1-PCA
Accuracy	0.74	0.64	**0.90**	0.74	**0.87**	0.67
Precision	0.81	0.66	**0.90**	0.74	**0.89**	0.67
Recall	0.93	0.96	**0.96**	0.93	**0.96**	1.0
MCC	0.55	−0.11	**0.78**	0.36	**0.74**	0

*Bold values indicate performance metrics for recommended, best-performing models.*

**TABLE 4:** Classification model performance (calculated with LOO cross-validation) for models trained with features from Feature Set 2.

	MARS		RFs		GPR	
	FS2-MARS	FS2-PCA	FS2-RF	FS2-PCA	FS2-GPR	FS2-PCA
Accuracy	0.62	0.67	**0.84**	0.67	0.69	0.67
Precision	0.68	0.68	**0.82**	0.73	0.70	0.67
Recall	0.82	0.93	**0.96**	0.79	0.93	1.0
MCC	0.04	0.11	**0.62**	0.22	0.23	0

*Bold values indicate performance metrics for recommended, best-performing models.*

Similar to those generated from Feature Set 1, the model-selected feature sets for Feature Set 2 resulted in a better performance than the PCs. This indicates that while the PCs successfully explain the variance in the data, they fail to accurately characterize the relationship between the features and the cardiomyocyte content. When only the features up to dd5 are considered, RFs most successfully predict if the CM content will be *sufficient*. The decrease in the performance for GPR models is possibly due to the removal of the dd7 average value of the DO concentration gradient. This dd7 feature was identified as relevant for predicting dd10 CM content using a GPR and could be an indicator of levels of cell metabolism.

Table 5 contains examples of bioreactor experiments and the predictions for those experiments given by GPR models using FS1-GPR, as well as the values of the bioprocess features indicated by the GPR model to be relevant. For some of the experiments with different prediction types, the feature values are quite similar, for example, the *“preculture time”* of experiments 36 and 26. However, for some experiments with the same prediction, the features have a wide range of values, for example, the *“dd2 cell density normalized DO gradient”* of experiments 16 and 26. These disparities indicate that the individual features alone are not sufficient to determine what will make a good or bad prediction and that all the selected features need to be considered as a whole.

**TABLE 5:** Examples of selected features from GPR-FS1.

Exp. No.	Prediction	Average DO concentration gradient dd2	Average cell density normalized DO concentration gradient dd2	Preculture Time [h]	Average DO concentration gradient dd7	dd0–dd1 cell density gradient	dd0 average pH gradient	Average cell density normalized DO concentration gradient dd5
3	TP	2.06	1.96	53	–3.23	0.02	–0.22	–6.06
40	TP	–1.01	–0.49	48	–1.71	1.34	–0.18	–3.08
16	FP	1.08	0.58	46	0.63	0.41	–0.21	–1.54
26	FP	–3.07	–1.83	48	–0.34	0.34	–0.03	–1.77
21	TN	–1.40	–1.06	47	0.17	0.29	–0.03	–1.75
42	TN	–1.13	–0.60	48	–0.22	0.62	–0.01	–2.03
36	FN	–0.76	–0.61	48	–6.51	1.60	–0.05	–3.48
	**Minimum**	–80.07	–47.24	45	–6.51	–0.76	–0.23	–17.33
	**Maximum**	14.37	7.00	56	9.61	2.63	0.08	3.73

For model selection purposes, the MCC gives the most important information about how the models perform, as it gives a measure of the correlation between the predicted and actual classes, similar to an R^2^ coefficient for a regression model. The other performance metrics should be assessed for their importance based on what the experimental goal of the bioprocess is. For example, if the differentiation process is being studied primarily for data collection and evaluating the outcomes, then maximizing the number of experimental datapoints being retained becomes more important, meaning that the precision of the model needs to be prioritized. However, for another application, such as an *in vivo* study, it would be more beneficial to stop unsuccessful experiments and start over, meaning that recall and accuracy of the model in identifying which experiments would not produce high CM contents would be prioritized. RF and GPR models were confirmed to be the most predictive of the dd10 cardiomyocyte content because their MCC values (around 0.80) and their accuracy and precisions of about 90% were higher than the other models investigated.

After testing how the models performed on the original dataset, their performance was evaluated using the test data. The test data consisted of data from the 16 processes that were not used for feature selection and model construction. The values of the selected features from those 16 “control processes” were used as inputs to make predictions of the final CM classification employing the trained models (Figure 1 Prediction by Classification), and those predictions were compared to the actual classifications from the process data. Since MARS had the worst performance for both feature sets, it was excluded from the analysis. The results are presented in Table 6. RFs and GPR had a similar performance for the test data for feature set FS1-GPR, both with an accuracy of 89%, precision and recall near 90%, and MCC values of 0.72. However, for the sets selected from feature set 2, RFs outperformed GPR. The results obtained for the test data are comparable to those for the data the models were trained on with LOO cross-validation, indicating that the models accurately captured the relationship between the features and the CM content necessary to make the classifications, while avoiding overfitting.

**TABLE 6:** Model performance on test data.

	RFs		GPR	
	FS1-RF	FS2-RF	FS1-GPR	FS2-GPR
Accuracy	**0.89**	**0.83**	0.83	0.72
Precision	**0.92**	**0.81**	0.92	0.72
Recall	**0.92**	**1.0**	0.85	1
MCC	**0.72**	**0.57**	0.61	0.11

*Bold values indicate performance metrics for recommended, best-performing models.*

The “*IWP2 treatment time*” feature was consistently chosen as having high importance for the prediction of the CM content. This feature describes the amount of time that the IWP2 molecule was allowed to remain in the bioreactor system, i.e., impact the differentiation process. However, this feature was only modulated for a fraction of the process runs and held constant at exactly 48 h for the rest. To evaluate if our models were able to classify the CM content without using that feature, an additional dataset was constructed. This data set was thus exclusively derived from the original set of 58 processes using only those process runs where the time of IWP2 presence was held constant at 48 h, and the “*IWP2 treatment time*” feature was excluded in the analysis. Since RFs performed well for all the previously considered feature sets, the performance was only evaluated using this model. LOO cross-validation and Monte Carlo cross-validation were used to calculate the performance metrics. The Monte Carlo cross-validation used a test set size of 5 and 40 Monte Carlo trials. The results are summarized in Table 7. It is thus worth highlighting that, when the IWP2 feature is removed, RFs still successfully predict *insufficient* CM content with comparable performances for both LOO and Monte Carlo cross-validation for all the feature sets.

**TABLE 7:** Model performance with constant IWP2 time.

	FS1-RF		FS2-RF	
	LOO	Monte Carlo	LOO	Monte Carlo
Accuracy	0.90	0.90	0.92	0.85
Precision	0.91	0.90	0.91	0.86
Recall	1.0	0.93	0.95	0.93
MCC	0.90	0.82	0.84	0.75

## Conclusion

In this paper, we have constructed data-driven models for prediction of the CM content on dd10 of hPSC differentiation processes, using existing data sets from bioreactor-based experiments. Using features up to dd7, we were able to identify if an experiment would have an *insufficient* final CM content of less than 90% with 90% accuracy and >90% precision with both RF and GPR models. Furthermore, we were able to identify if an experiment would have an *insufficient* final CM content on dd5 with 84% accuracy with a RF model. Through feature selection methods, these predictions used less than 16% of the collected data, potentially reducing the amount of resource-intense manual collection of data. Although these models can accurately and precisely predict final CM content, they do not provide any insight into the overall quantity of CMs produced or the resulting functionality and maturity of these cells. In addition, the prediction models were only constructed for small set of data with limited ranges of all the features. However, the ability to model the outcome of differentiation experiments at an early stage of differentiation, enables the timely interruption of failing experiments, providing savings in both time and resources. More importantly, results from the study provide valuable hypotheses for further experiments to improve robustness and reproducibility of cardiac differentiation processes, and have the potential to be leveraged for a broad variety of hPSC-derived cell and tissue production experiments.

## Data Availability Statement

The datasets generated for this study can be found in the online repositories. The names of the repository/repositories and accession number(s) can be found below: The datasets will be made available in the Cremaschi Group Github repository (https://github.com/CremaschiLab/Cardiac_Differentiation_Modeling).

## Author Contributions

BW performed all of the computational analysis and model development tasks, including the construction of the feature sets, feature selection, classification model construction, and wrote the related sections of the manuscript. CH and WL performed the experimental work, with assistance from FM. KR, CH, and WL collected and organized the experimental data into an appropriate database. RZ, CH, WL, EL, and FF gave input on biological implications of the results from the computational analysis. The study was conceptualized by SC, EL, RZ, and CH. SC, EL, and RZ provided supervision and guidance for the work. All authors contributed to manuscript drafting, revisions, and read and approved the submitted version.

## Conflict of Interest

The authors declare that the research was conducted in the absence of any commercial or financial relationships that could be construed as a potential conflict of interest.
