# Supplementary material for: Prediction of Human Induced Pluripotent Stem Cell Cardiac Differentiation Outcome by Multifactorial Process Modeling
Source: Front Bioeng Biotechnol. 2020 Jul 23;8:851. doi: 10.3389/fbioe.2020.00851 (PMC7390976; doi:10.3389/fbioe.2020.00851)
Supplement: Supplementary file 1 [file Data_Sheet_1.pdf]

Supplementary Material

1 Supplementary Tables

Supplemental Table 1. Summary of Feature Selection Results for Feature Set 1 (**X** = selected). The minimum and maximum value as well as the Pearson and Spearman correlation with the dd10 cardiomyocyte content is given.

Feature	Minimum	Maximum	Pearson	Spearman	FS1-PCA	FS1-MARS	FS1-RF	FS1-GPR
dd0 Cell Density	0.30	1.28	0.05	0.04		X		
dd0-dd1 Cell Density Gradient	-0.76	2.63	0.13	0.28				X
dd1 Cell Density	0.29	1.36	0.50	0.54			X	
dd1-dd2 Cell Density Gradient	0.03	3.57	-0.37	-0.01				
dd2 Cell Density	0.35	2.40	0.06	0.24				
dd2-dd3 Cell Density Gradient	-0.46	4.13	-0.30	-0.18				
dd3 Cell Density	0.69	3.00	-0.18	-0.07				
dd3-dd5 Cell Density Gradient	-0.49	0.81	0.38	0.31		X		
dd5 Cell Density	0.68	3.06	0.31	0.25				
dd5-dd7 Cell Density Gradient	-0.79	0.40	0.17	0.22				
dd7 Cell Density	0.16	3.05	0.44	0.54			X	X
dd0 Aggregate Size	71.49	148.91	0.37	0.05	X	X		
dd0-dd1 Aggregate Size Gradient	-0.03	0.65	-0.51	-0.38				
dd1 Aggregate Size	79.61	199.12	0.05	-0.06	X			
dd1-dd2 Aggregate Size Gradient	-0.19	0.52	-0.05	0.06				
dd2 Aggregate Size	121.07	177.55	0.12	0.15	X			
dd2-dd3 Aggregate Size Gradient	-0.25	0.46	-0.13	-0.18				
dd3 Aggregate Size	114.85	219.12	-0.08	-0.16	X			
dd3-dd5 Aggregate Size Gradient	-0.22	3.26	-0.20	0.08				
dd5 Aggregate Size	115.77	754.75	-0.23	-0.02	X			
dd7 Aggregate Size	106.29	439.52	0.03	0.21	X			
dd5-dd7 Aggregate Size Gradient	-0.55	0.50	0.35	0.29			X	
Preculture Time [h]	45.00	56.00	-0.18	-0.17				X

Supplemental Table 1. Summary of Feature Selection Results for Feature Set 1 (**X** = selected). The minimum and maximum value as well as the Pearson and Spearman correlation with the dd10 cardiomyocyte content is given.

Feature	Minimum	Maximum	Pearson	Spearman	FS1-PCA	FS1-MARS	FS1-RF	FS1-GPR
Start Preculture Perfusion [h after inoc] d1-d2	25.00	28.50	-0.51	-0.65				
IWP2 Treatment Time [h]	43.00	54.00	0.09	0.34		X		
Average DO concentration d0	19.40	155.99	0.07	0.18	X			
Average DO concentration d1	20.01	146.91	0.07	0.27	X			
Average DO concentration dd0	19.97	144.99	0.06	0.18	X		X	
Average DO concentration dd1	19.89	143.00	0.12	0.23	X			
Average DO concentration dd2	27.27	117.99	-0.09	-0.12	X	X		
Average DO concentration dd3	19.68	126.34	0.01	0.00	X			
Average DO concentration dd4	19.94	197.68	0.14	0.14	X			
Average DO concentration dd5	19.03	237.49	0.03	-0.05	X			
Average DO concentration dd6	22.74	237.25	-0.17	-0.38	X			
Average DO concentration dd7	14.14	214.26	-0.23	-0.45	X			
Average DO concentration gradient d0	-355.00	238.56	0.31	0.40	X		X	
Average DO concentration gradient d1	-12.36	69.65	0.07	-0.04	X			
Average DO concentration gradient dd0	-5.80	2.76	0.00	0.03				
Average DO concentration gradient dd1	-7.14	3.15	0.01	-0.01				
Average DO concentration gradient dd2	-80.07	14.37	-0.14	-0.32				X
Average DO concentration gradient dd3	-4.23	8.50	0.14	0.11	X			

Supplemental Table 1. Summary of Feature Selection Results for Feature Set 1 (**X** = selected). The minimum and maximum value as well as the Pearson and Spearman correlation with the dd10 cardiomyocyte content is given.

Average DO concentration gradient dd4	-52.10	3.47	-0.28	-0.34		X		
Average DO concentration gradient dd5	-28.23	3.96	-0.03	-0.20	X			
Average DO concentration gradient dd6	-38.29	-2.38	0.02	-0.04	X		X	
Average DO concentration gradient dd7	-6.51	9.61	-0.17	0.11	X			X
Cell density normalized DO concentration dd0	26.10	325.81	-0.25	-0.27	X			
Cell density normalized DO concentration dd1	21.51	242.12	-0.27	-0.26	X			
Cell density normalized DO concentration dd2	21.77	110.53	-0.14	-0.11	X			
Cell density normalized DO concentration dd3	15.29	95.74	-0.05	0.02	X			
Cell density normalized DO concentration dd5	11.07	236.31	-0.16	-0.33	X			
Cell density normalized DO concentration dd7	6.69	325.13	-0.16	-0.58	X		X	
Average cell density normalized DO gradient dd0	-5.66	3.10	0.09	0.05				X
Average cell density normalized DO gradient dd1	-5.00	3.59	0.10	0.08				
Average cell density normalized DO gradient dd2	-47.24	7.00	-0.10	-0.21			X	X
Average cell density normalized DO gradient dd3	-3.82	7.20	0.07	0.10	X			
Average cell density normalized DO gradient dd5	-17.33	3.73	0.13	-0.15	X			X

Supplemental Table 1. Summary of Feature Selection Results for Feature Set 1 (**X** = selected). The minimum and maximum value as well as the Pearson and Spearman correlation with the dd10 cardiomyocyte content is given.

Average cell density normalized DO gradient dd7	-7.23	18.30	-0.12	0.13	X		X	
dd0 Average acceleration of DO gradient	-2114.51	1270.22	-0.19	-0.30	X			
dd1 Average acceleration of DO gradient	-548.72	159.65	-0.26	-0.23	X			
dd2 Average acceleration of DO gradient	-717.88	569.36	-0.15	-0.13	X			
dd3 Average acceleration of DO gradient	-1403.97	6.14	-0.16	-0.26	X			
dd5 Average acceleration of DO gradient	-408.35	1810.40	0.18	0.12	X			
dd7 Average acceleration of DO gradient	-727.09	281.59	-0.19	-0.10	X			
dd0 Average acceleration of cell density normalized DO gradient	-3410.50	2190.03	-0.14	-0.37	X		X	
dd1 Average acceleration of cell density normalized DO gradient	-518.56	190.06	-0.25	-0.21	X			
dd2 Average acceleration of cell density normalized DO gradient	-487.08	353.64	-0.14	-0.11	X			
dd3 Average acceleration of cell density normalized DO gradient	-684.86	8.96	-0.14	-0.26	X			
dd5 Average acceleration of cell density normalized DO gradient	-296.53	1149.46	0.19	0.11	X			
dd7 Average acceleration of cell density normalized DO gradient	-635.01	268.18	-0.19	-0.10	X			
Overall Average pH	6.57	7.23	-0.14	-0.05				

Supplemental Table 1. Summary of Feature Selection Results for Feature Set 1 (**X** = selected). The minimum and maximum value as well as the Pearson and Spearman correlation with the dd10 cardiomyocyte content is given.

Overall density gradient	-0.80	4.65	0.24	0.37		X		
Overall aggregate size gradient	-0.03	2.99	-0.19	0.07				
d0 Average pH	6.86	7.26	0.04	-0.14				
d0 Average pH Gradient	-0.18	0.01	0.21	-0.06			X	
d1 Average pH	6.46	7.86	-0.12	-0.21				
d1 Average pH Gradient	-0.21	0.01	-0.09	-0.01		X		
dd0 Average pH	6.37	7.73	0.16	0.26				
dd0 Average pH Gradient	-0.23	0.08	-0.17	-0.25				X
dd1 Average pH	5.15	7.87	-0.09	-0.11		X		
dd1 Average pH Gradient	-0.26	0.26	0.20	-0.02				
dd2 Average pH	5.99	7.31	-0.42	-0.46		X	X	
dd2 Average pH Gradient	-0.18	0.01	0.51	0.49				
dd3 Average pH	6.50	7.36	-0.27	-0.16				
dd3 Average pH Gradient	-0.17	0.02	0.25	0.12			X	
dd4 Average pH	6.09	7.24	-0.12	0.06				
dd4 Average pH Gradient	-0.18	0.01	0.26	0.24				
dd5 Average pH	6.56	8.25	0.09	0.10				
dd5 Average pH Gradient	-0.29	0.02	0.15	0.10				
dd6 Average pH	6.39	7.25	-0.23	-0.12				
dd6 Average pH Gradient	-0.11	0.02	-0.02	0.21				
dd7 Average pH	6.74	7.31	0.23	0.20				X
dd7 Average pH Gradient	-0.16	0.02	0.34	0.08				
dd0 Lactate Concentration	11.59	15.83	0.31	0.33				
dd1 Lactate Concentration	9.05	19.59	0.10	0.19	X			
dd3 Lactate Concentration	1.00	18.73	0.07	0.19				
dd5 Lactate Concentration	10.46	20.38	0.10	0.04	X			
dd7 Lactate Concentration	5.94	20.59	-0.02	-0.03				

Supplemental Table 1. Summary of Feature Selection Results for Feature Set 1 (**X** = selected). The minimum and maximum value as well as the Pearson and Spearman correlation with the dd10 cardiomyocyte content is given.

dd0 Glucose Concentration	7.14	11.95	-0.24	-0.16				
dd1 Glucose Concentration	0.60	6.20	0.20	0.10				
dd3 Glucose Concentration	0.00	10.27	-0.08	-0.21				
dd5 Glucose Concentration	0.00	6.91	0.03	0.05				
dd7 Glucose Concentration	0.00	8.33	-0.14	-0.23				

Supplemental Table 2. Summary of Feature Selection Results for Feature Set 2 (**X** = selected). The minimum and maximum value as well as the Pearson and Spearman correlation with the dd10 cardiomyocyte content is given.

	**Minimum**	**Maximum**	**Pearson**	**Spearman**	**FS2-PCA**	**FS2-MARS**	**FS2-RF**	**FS2-GPR**
dd0 Cell Density	0.30	1.28	0.05	0.04		X		
dd0-dd1 Cell Density Gradient	-0.76	2.63	0.13	0.28				X
dd1 Cell Density	0.29	1.36	0.50	0.54				
dd1-dd2 Cell Density Gradient	0.03	3.57	-0.37	-0.01				
dd2 Cell Density	0.35	2.40	0.06	0.24				
dd2-dd3 Cell Density Gradient	-0.46	4.13	-0.30	-0.18				
dd3 Cell Density	0.69	3.00	-0.18	-0.07				
dd3-dd5 Cell Density Gradient	-0.49	0.81	0.38	0.31		X		
dd5 Cell Density	0.68	3.06	0.31	0.25				
dd0 Aggregate Size	71.49	148.91	0.37	0.05	X	X		
dd0-dd1 Aggregate Size Gradient	-0.03	0.65	-0.51	-0.38				
dd1 Aggregate Size	79.61	199.12	0.05	-0.06	X			
dd1-dd2 Aggregate Size Gradient	-0.19	0.52	-0.05	0.06				
dd2 Aggregate Size	121.07	177.55	0.12	0.15	X		X	
dd2-dd3 Aggregate Size Gradient	-0.25	0.46	-0.13	-0.18			X	
dd3 Aggregate Size	114.85	219.12	-0.08	-0.16	X			
dd3-dd5 Aggregate Size Gradient	-0.22	3.26	-0.20	0.08				
dd5 Aggregate Size	115.77	754.75	-0.23	-0.02	X			
Preculture Time [h]	45.00	56.00	-0.18	-0.17				
Start Preculture Perfusion [h after inoc] d1-d2	25.00	28.50	-0.51	-0.65				
Presence of IWP2 [h]	43.00	54.00	0.09	0.34		X		X
Average DO concentration d0	19.40	155.99	0.07	0.18	X		X	
Average DO concentration d1	20.01	146.91	0.07	0.27	X			
Average DO concentration dd0	19.97	144.99	0.06	0.18	X		X	

Supplemental Table 2. Summary of Feature Selection Results for Feature Set 2 (**X** = selected). The minimum and maximum value as well as the Pearson and Spearman correlation with the dd10 cardiomyocyte content is given.

Average DO concentration dd1	19.89	143.00	0.12	0.23	X			
Average DO concentration dd2	27.27	117.99	-0.09	-0.12	X	X		
Average DO concentration dd3	19.68	126.34	0.01	0.00	X			
Average DO concentration dd4	19.94	197.68	0.14	0.14	X		X	
Average DO concentration dd5	19.03	237.49	0.03	-0.05	X			
Average DO concentration gradient d0	-355.00	238.56	0.31	0.40	X		X	
Average DO concentration gradient d1	-12.36	69.65	0.07	-0.04	X			
Average DO concentration gradient dd0	-5.80	2.76	0.00	0.03				
Average DO concentration gradient dd1	-7.14	3.15	0.01	-0.01				
Average DO concentration gradient dd2	-80.07	14.37	-0.14	-0.32			X	X
Average DO concentration gradient dd3	-4.23	8.50	0.14	0.11				
Average DO concentration gradient dd4	-52.10	3.47	-0.28	-0.34		X	X	
Average DO concentration gradient dd5	-28.23	3.96	-0.03	-0.20	X		X	
Cell density normalized DO concentration dd0	26.10	325.81	-0.25	-0.27	X			

Supplemental Table 2. Summary of Feature Selection Results for Feature Set 2 (**X** = selected). The minimum and maximum value as well as the Pearson and Spearman correlation with the dd10 cardiomyocyte content is given.

Cell density normalized DO concentration dd1	21.51	242.12	-0.27	-0.26	X		X	
Cell density normalized DO concentration dd2	21.77	110.53	-0.14	-0.11	X			
Cell density normalized DO concentration dd3	15.29	95.74	-0.05	0.02	X			
Cell density normalized DO concentration dd5	11.07	236.31	-0.16	-0.33	X			
Average cell density normalized DO gradient dd0	-5.66	3.10	0.09	0.05				
Average cell density normalized DO gradient dd1	-5.00	3.59	0.10	0.08				
Average cell density normalized DO gradient dd2	-47.24	7.00	-0.10	-0.21				
Average cell density normalized DO gradient dd3	-3.82	7.20	0.07	0.10				
Average cell density normalized DO gradient dd5	-17.33	3.73	0.13	-0.15				X
dd0 Average acceleration of DO gradient	-2114.51	1270.22	-0.19	-0.30	X			
dd1 Average acceleration of DO gradient	-548.72	159.65	-0.26	-0.23	X			
dd2 Average acceleration of DO gradient	-717.88	569.36	-0.15	-0.13	X			X
dd3 Average acceleration of DO gradient	-1403.97	6.14	-0.16	-0.26	X			
dd5 Average acceleration of DO gradient	-408.35	1810.40	0.18	0.12	X		X	

Supplemental Table 2. Summary of Feature Selection Results for Feature Set 2 (**X** = selected). The minimum and maximum value as well as the Pearson and Spearman correlation with the dd10 cardiomyocyte content is given.

dd0 Average acceleration of cell density normalized DO gradient	-3410.50	2190.03	-0.14	-0.37	X			
dd1 Average acceleration of cell density normalized DO gradient	-518.56	190.06	-0.25	-0.21	X		X	
dd2 Average acceleration of cell density normalized DO gradient	-487.08	353.64	-0.14	-0.11	X		X	
dd3 Average acceleration of cell density normalized DO gradient	-684.86	8.96	-0.14	-0.26	X			
dd5 Average acceleration of cell density normalized DO gradient	-296.53	1149.46	0.19	0.11	X			X
Overall Average pH	6.57	7.23	-0.15	-0.08				
Overall density gradient	-0.80	4.65	0.24	0.37		X		
Overall aggregate size gradient	-0.03	2.99	-0.19	0.07			X	
d0 Average pH	6.86	7.26	0.04	-0.14				
d0 Average pH Gradient	-0.18	0.01	0.21	-0.06			X	
d1 Average pH	6.46	7.86	-0.12	-0.21				
d1 Average pH Gradient	-0.21	0.01	-0.09	-0.01		X		
dd0 Average pH	6.37	7.73	0.16	0.26				
dd0 Average pH Gradient	-0.23	0.08	-0.17	-0.25				
dd1 Average pH	5.15	7.87	-0.09	-0.11		X	X	
dd1 Average pH Gradient	-0.26	0.26	0.20	-0.02				
dd2 Average pH	5.99	7.31	-0.42	-0.46		X		
dd2 Average pH Gradient	-0.18	0.01	0.51	0.49			X	
dd3 Average pH	6.50	7.36	-0.27	-0.16				

Supplemental Table 2. Summary of Feature Selection Results for Feature Set 2 (**X** = selected). The minimum and maximum value as well as the Pearson and Spearman correlation with the dd10 cardiomyocyte content is given.

dd3 Average pH Gradient	-0.17	0.02	0.25	0.12				
dd4 Average pH	6.09	7.24	-0.12	0.06				
dd4 Average pH Gradient	-0.18	0.01	0.26	0.24				X
dd5 Average pH	6.56	8.25	0.09	0.10				
dd5 Average pH Gradient	-0.29	0.02	0.15	0.10				
dd0 Lactate Concentration	11.59	15.83	0.31	0.33				
dd1 Lactate Concentration	9.05	19.59	0.10	0.19	X			
dd3 Lactate Concentration	1.00	18.73	0.07	0.19				
dd5 Lactate Concentration	10.46	20.38	0.10	0.04				
dd0 Glucose Concentration	7.14	11.95	-0.24	-0.16				
dd1 Glucose Concentration	0.60	6.20	0.20	0.10				
dd3 Glucose Concentration	0.00	10.27	-0.08	-0.21				
dd5 Glucose Concentration	0.00	6.91	0.03	0.05				

Supplementary Table 3. FS1-MARS

Feature	Importance	Pearson	Spearman
dd0-dd1 cell density gradient	27%	0.13	0.28
IWP2 Treatment Time [h]	20%	-0.50	-0.65
dd5-dd7 cell density gradient	20%	0.35	0.29
d0 DO concentration gradient	12%	0.31	0.40
dd3 Cell density normalized DO concentration	8.2%	0.04	0.03
dd3 aggregate size	6.9%	-0.08	-0.16
dd5 average pH	0.03%	0.09	0.10
dd3 Acceleration of cell density normalized DO gradient	1.8%	-0.14	-0.26
dd1 aggregate size	1.7%	0.05	-0.06
dd2 Cell density normalized DO concentration	1.1%	-0.41	-0.46
dd1 cell density	0.07%	0.50	0.54
dd3 Average acceleration of DO gradient	0.07%	-0.16	-0.26

Supplementary Table 4. FS1-RF

Features	Importance	Pearson	Spearman
dd5-dd7 Aggregate Size Gradient	16%	0.35	0.29
dd1 Cell Density	14%	0.50	0.54
Average cell density normalized DO gradient dd2	13%	-0.10	-0.21
d0 Average pH Gradient	13%	0.21	-0.06
Average cell density normalized DO gradient dd7	8.7%	-0.12	0.13
dd2 Average pH	7.5%	-0.42	-0.46
Average DO concentration dd0	6.9%	0.06	0.18
Cell density normalized DO concentration dd7	5.3%	-0.16	-0.58
Average DO concentration gradient dd6	3.3%	0.02	-0.04
Average DO concentration gradient d0	3.0%	0.31	0.40
dd3 Average pH Gradient	3.0%	0.25	0.12
dd7 Cell Density	3.0%	0.44	0.54
dd0 Average acceleration of cell density normalized DO gradient	2.7%	-0.14	-0.37

Supplementary Table 5. FS1-GPR

Feature	Sensitivity	Pearson	Spearman
Average DO concentration gradient dd2	8299.80	-0.14	-0.32
dd2 average cell density normalized DO gradient	5787.15	-0.10	-0.21
Preculture Time [h]	2256.94	-0.18	-0.17
Average DO concentration gradient dd7	790.92	-0.17	0.11
dd0-dd1 Cell Density Gradient	70.18	0.13	0.28
dd0 Average pH Gradient	15.94	-0.17	-0.25
dd5 average cell density normalized DO gradient	8.34	0.13	-0.15
dd0 average cell density normalized DO gradient	2.50	0.09	0.05
dd7 Cell Density	0.34	0.44	0.54
dd7 Average pH	0.09	0.23	0.20

Supplementary Table 6. FS2-RF

Feature	Importance	Pearson	Spearman
d0 DO concentration gradient	17%	0.31	0.40
dd2 average pH gradient	11%	0.51	0.49
dd0 average DO	11%	0.06	0.18
dd1 average pH	9.3%	-0.09	-0.11
dd1 average cell density normalized DO concentration	7.4%	-0.27	-0.26
d0 average DO concentration	5.5%	0.07	0.18
Total aggregate size gradient	4.1%	0.09	0.10
dd2 DO concentration gradient	4.0%	-0.14	-0.32
dd4 average DO concentration	3.9%	0.14	0.14
dd5 DO concentration gradient	3.6%	-0.03	-0.20
dd2-dd3 aggregate size gradient	2.6%	-0.13	-0.18
dd2 aggregate size	2.4%	0.12	0.15
dd1 Average acceleration of cell density normalized DO gradient	2.4%	-0.25	-0.21
dd4 DO concentration gradient	2.2%	-0.28	-0.34
dd5 Average acceleration of DO gradient	2.2%	0.18	0.12
dd2 Average acceleration of cell density normalized DO gradient	2.2%	-0.14	-0.11

Supplementary Table 7. FS2-MARS

Features	Importance	Pearson	Spearman
IWP2 Treatment Time [h]	30%	0.09	0.34
dd2 average DO Concentration	24%	-0.09	-0.12
dd2 average pH	21%	-0.42	-0.46
d1 average pH gradient	17%	-0.09	-0.01
dd0 cell density	3.2%	0.05	0.04
Total cell density gradient	2.7%	0.12	0.12
dd4 DO concentration gradient	0.9%	-0.28	-0.34
dd0 aggregate size	0.8%	0.37	0.05
dd1 average pH	0.8%	-0.09	-0.11

dd3-dd5 cell density gradient	0.2%	0.38	0.31

Supplementary Table 8. FS2-GPR

Features	Sensitivity	Pearson	Spearman
IWP2 Treatment Time [h]	21	0.09	0.34
dd2 Average DO concentration gradient	19	-0.14	-0.32
dd5 Cell density normalized DO concentration	3.5	-0.15	-0.16
dd0-dd1 cell density gradient	0.84	0.13	0.28
dd4 average pH gradient	0.67	0.26	0.24
dd5 Average acceleration of cell density normalized DO gradient	0.17	0.19	0.11
dd2 Average acceleration of DO gradient	0.12	-0.15	-0.13

2 Extended Machine Learning Technique Descriptions

2.1 Machine learning techniques

2.1.1 Multivariate adaptive regression splines

Multivariate adaptive regression splines (MARS) models are nonparametric statistical models that consist of a linear summation of basis functions (Friedman, 1991). In general, basis functions are either a constant, a hinge function, or the product of two or more hinge functions. Eq. (3) demonstrates a hinge function.

$$f(x) = \max(0, c - x) \qquad \text{Eq. (3)}$$

where c is a constant value, which is typically called a "knot", and x represents a model input variable. According to Eq. (3), the function value is equal to zero for all the input values less than c and is equal to $c - x$ otherwise.

MARS models are built in two steps, with the first step continuously adding basis functions and overfitting the model to the training data (Friedman, 1991). The training data are the set of input/output points which are used to train a model to predict or classify. The second step is a backward pruning pass, where the terms with little effect on the model estimation are removed from the model based on a generalized cross-validation criterion (Friedman, 1991). Cross-validation is a method to evaluate the result statistically and determine if it is generalizable (Burman, 1989).

2.1.2 Random forests

Random forests (RFs) are a machine learning method that utilizes a set of decision trees for predicting an output based on input data. Each tree is built independently based on a random subspace of the training data. The final output of a random forest model is determined by averaging the output value of every tree in the forest (Breiman, 2001). The features are selected according to the importance level calculated by the random forest model. The importance level is based on the impact of a feature on improving the separation of the data in each decision node of the tree.

2.1.3 Gaussian process regression

Gaussian process regression (GPR) is a nonparametric machine learning method where the prediction of the output corresponding to an unknown input is calculated based on a weighted average of outputs for known inputs using a similarity metric. The similarity metric is defined by a kernel function (Rasmussen and Williams, 2005). The kernel function used for all GPR models in this paper is a radial basis function.

GPR can be used for feature selection with its built-in automatic relevance determination (ARD) method. Further sensitivity analysis (Eq. (4)) on the ARD results (Blix and Eltoft, 2018) provides an even greater separation of the features for selection. The empirical estimate of the sensitivity of the GPR model output to the jth feature is calculated by:

$$\hat{s}_j = \frac{1}{N}\sum_{n=1}^{N}\left(\frac{\partial\phi(x^{(n)})}{\partial x_j}\right)^2 \qquad \text{Eq. (4)}$$

where $\phi(x)$ is the mean function of the GPR model and N is the number of data points used in training the model.

3 Examples of the Average acceleration of the (cell density normalized) DO gradient

Supplementary Fig. 1 shows the range of values for the feature "*average acceleration of the cell density normalized DO gradient*" during the differentiation phase for both the experiments with sufficient and insufficient cardiomyocyte contents. This feature was only selected by our recommended models for differentiation days 0,1,2, and 5. There is no significant difference in the ranges of the the values of the for this feature between the experiments that had sufficient CM content and the ones that had insufficient CM content. These individual features considered by themselves are not sufficient to determine the final outcome of the experiment, which is why we consider all of the features as a whole with machine learning technqiues.

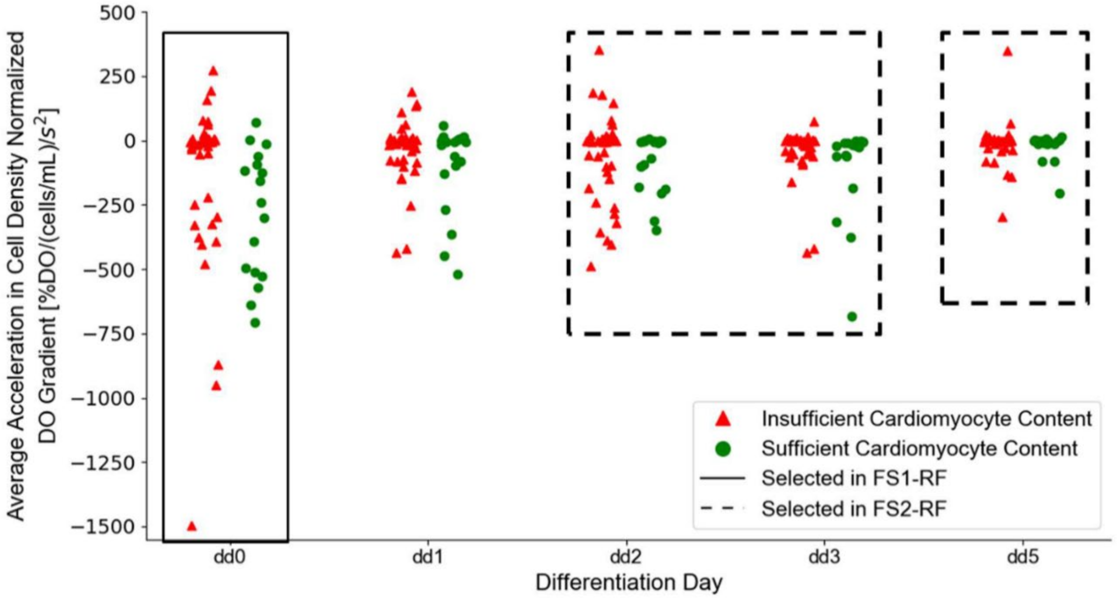

Supplementary Figure 1 – Acceleration of the cell density normalized DO gradient averaged by differentiation day.
